# Trials, Tribulations, and Triumphs: Research and Publishing From the Undergraduate Perspective

**DOI:** 10.3389/fpsyg.2018.02411

**Published:** 2018-12-03

**Authors:** Sarah J. Matthews, Marissa N. Rosa

**Affiliations:** Department of Psychology, Southwestern University, Georgetown, TX, United States

**Keywords:** undergraduate research, student perceptions, publishing, best practices in teaching, undergraduate publication

## Introduction

Demanding, engaging, and yes–frustrating at times–conducting research as an undergraduate can be an incredibly transformative personal and academic experience. As members of a year-long senior-level research lab in social psychology at a primarily undergraduate institution, we are grateful for the opportunity to have participated in all phases of the research process, including publication. Having less structure and requiring more involvement than many college courses, conducting high-level research comes with unique challenges and benefits. As such, we are excited to offer our perspective on these obstacles and rewards, along with specific tips for research instructors based on how our lab successfully produced publishable research.

## Background on our lab

Our research lab consisted of five undergraduate research assistants working with one faculty research advisor. For best practices on engaging undergraduates in publishable research from her perspective as an instructor, see Giuliano (2019a,b). Research assistants were invited by our advisor to collaborate in her lab based on our performance in previous classes (e.g., a year-long research methods course that required complete APA paper write-ups on four projects: one experimental replication plus original observational, correlational, and 2 × 2 experimental projects). Our lab met once a week for two and a half hours under the supervision of our lab instructor, and we were expected to work an average of 8 to 10 hours per week outside of meetings (although we often worked more) on tasks such as searching the literature, generating hypotheses, designing studies, collecting data, analyzing data, and writing up the results. Assessment for each semester was a letter grade, and As were not automatic (i.e., they had to be earned). The expectation of our instructor's lab was that we work at a much more advanced level than during our research methods class, that we write multiple manuscript drafts, and that with good results, we would present our work at conferences and submit it for publication to a non-undergraduate refereed journal.

## Challenges of research

From the beginning, working collaboratively on such an intensive project posed considerable challenges. Our research group was comprised of diverse personality types, and we had to learn to communicate in ways that were effective, yet respectful, in order to advance our project while maintaining group rapport. Speaking up when we felt it was necessary, especially when disagreeing with another's opinion, proved to be an uncomfortable yet essential lesson. We also had to learn to trust our own judgment and the judgment of other group members. We readily accepted criticism and constructive feedback in order to make progress, and we had to overcome procrastination and complacency in order to stay on course as a group. Academically, conducting research was much more “messy” than previous coursework anyone in our group had participated in; the lack of structure and increased ambiguity could be confusing and frustrating, and preventing tension within our lab became a priority.

Perhaps most importantly, our involvement in research did not end after our year together in the lab. Although our class project was complete, the process of publishing our findings provided a new set of challenges. Aside from no longer being together physically, we were juggling other responsibilities; graduation, graduate school and job applications, and post-graduate employment got in the way as we stumbled through the uncertainty of life in our early 20s. As such, new obstacles arose, including staying immersed in the research, staying in contact, and staying motivated without official meeting times. From a practical perspective, there was less access to resources such as SPSS software, online literature databases, and our lab instructor. Additionally, because of our distance, communication more often took the form of emails or phone calls rather than in-person meetings. As lead authors on our manuscripts, we found the continual process of revision and submission to be time consuming, and, in the face of rejection, occasionally disheartening. The time that elapsed between submitting work to a journal and receiving feedback (sometimes several months) created an “out of sight, out of mind” effect, which made returning to our manuscript and diving back into our work a motivational challenge.

## Benefits of research

Thankfully, the advantages of a rigorous research experience are numerous and far outweigh the challenges, and previous research substantiates the widespread benefits we experienced. For example, Russell et al. (2007) found that, after participating in undergraduate research opportunities, students reported increased understanding of how to conduct research and increased confidence in their skills as researchers. For us, completing a research project in social psychology improved our work ethic and transformed how we approached problems. Specifically, our research experience trained us to be diligent, critical thinkers who were capable of using our time efficiently by being responsible, independent, and proactive. These lessons led to arguably the most important benefit in line with previous research that each of us experienced–an increased confidence in ourselves and the work we produced.

In addition to becoming more confident and capable researchers, Russell et al. (2007) showed that participation in undergraduate research led to an increased awareness of what graduate schools are like. By participating in an experience normally reserved for graduate students, our undergraduate research experience provided us with a greater understanding of what to expect–and what will be expected of us–in graduate school, ultimately allowing us to clarify our future goals. In a similar vein, Hathaway et al. (2002) found that undergraduate research participation influences postgraduate aspirations and faculty relationships, as students who conducted research were more likely to pursue graduate education, more likely to pursue law, medical, or doctoral degrees, and more likely to ask faculty for job recommendations compared to students who did not conduct undergraduate research. Similarly, we experienced professional benefits such as the opportunity to present at conferences and the possibility of strong letters of recommendation from our advisor. The detail to which she can speak to our abilities as students is vital to us getting into graduate and postgraduate programs (including law school and medical school) that we are interested in pursuing. For those of us who decided not to pursue graduate school in psychology, the conscientiousness, responsibility, and perseverance that research requires prepared us for an easier transition to higher forms of education and post-graduation employment.

## Tips for success

Having experienced firsthand the challenges and benefits of research during our time as undergraduates, we are very proud of all that we accomplished (i.e., 3 national conference presentations, 2 publications, and 1 paper under review; see Appendix). As such, we wanted to share some of our specific practices that we believe contributed to our success as an undergraduate research group in hopes these tips may benefit other instructors in creating more productive research labs.

One of the most critical practices that we implemented was to motivate everyone to propose new ideas and to ask questions, which allowed our lab to function as a supportive, creative environment. Importantly, our lab instructor was extremely welcoming and encouraging. Her positivity and enthusiasm were critical to us seeking guidance when we became confused or insecure, therefore preventing us from falling behind and getting discouraged. However, as more introverted members of the group, both of us still found sharing ideas and questions difficult at times, despite the supportive environment. To combat this issue, our lab instructor often went around the room asking for each student's input. Although intimidating at first, this practice made everyone more comfortable speaking up during our lab meetings, as well as in our group conversations outside the lab. As the research process went on, we became more secure and confident in our thoughts and opinions.

In the beginning of our time as a research lab, each of us had difficulty using our time efficiently because we had a week between our scheduled class meetings. At our instructor's suggestion, we chose to address this problem by taking detailed notes during our weekly meetings that included everything that we discussed and that documented our progress. To avoid diffusion of responsibility (Latané and Darley, 1968), we also created a list of tasks to be completed before our next group meeting. At the end of each meeting, our to-do list was divided as we each volunteered to take on a number of tasks depending on our interests, skills, and the amount of time we had that week considering our course loads and other responsibilities (e.g., jobs, clubs, sports). The assignment of tasks led to a more even distribution of the workload and made us more responsible contributors because other group members could hold us accountable. Weekly progress reports (required by the instructor) were completed by each lab member to document how many hours we spent on our specific tasks, further contributing to a more fair and equitable division of our time. Taken together, these steps helped reduce social loafing, the phenomena in which individuals are less motivated to work hard on group tasks (Latané et al., 1979). Furthermore, our lab instructor continually stressed the potential opportunities we could experience if our research was successful (e.g., presenting at conferences and publishing our work). In doing so, she activated the collective effort model (Karau and Williams, 2001), as individuals in the group believed their efforts were meaningful in achieving our desired outcomes, another factor that reduces the likelihood of social loafing.

To use our time apart efficiently, our lab instructor asked us to first brainstorm research ideas and designs separately. Despite popular belief that brainstorming in groups is highly productive, research has shown that group brainstorming is actually less effective compared to group members brainstorming alone (Mullen et al., 1991). When asked to quickly brainstorm in a group setting without preparing beforehand, undergraduate research assistants may feel self-conscious and hesitant to share their thoughts. Consequently, being forced to generate a certain number of well-thought out ideas on our own proved helpful, especially at the beginning of our project when we used lab meetings to go over each student's ideas. Each student contributed suggestions for research topics, potential variables, and correlational and experimental designs, and we were able to build and expand on each others' original ideas to create the highest-quality project possible.

For the writing aspect of our research, our instructor had multiple methods for producing publication-quality papers. First, group members wrote their own individual papers, allowing for multiple conceptualizations of our research. Second, our instructor had us write our papers in sections according to where we were in the research process. For example, the first semester, we completed our literature review, designed the study, and coded our survey; thus, the introduction and methods of our paper were due before our winter break. (The second semester, we analyzed the data and wrote our results and discussion sections before going back to polish and revise our introduction and method sections from the previous semester.) As a result, all of the relevant information was fresh on our minds, and we were able to easily remember and discuss all the details within our papers. Third, we used a peer review process that allowed us to see how other group members discussed our project. By doing so, we were able to strengthen our own papers by incorporating what other group members had explained more clearly and effectively. On a more general note, our lab instructor stressed meticulousness and diligence in our writing (see Giuliano, 2019a,b). Thus, we always allotted more time to work on our paper than we thought was necessary to allow sufficient time for careful writing, rewriting, and proofreading of our papers. Thankfully, the time we dedicated to the writing of our research resulted in high-quality work that was accepted for presentation and publication.

## Conclusion

In closing, participating in an intensive, graduate-level research lab as undergraduates challenged us in ways that neither of us had been challenged before. However, through the process of finding out what worked for us under the guidance of our instructor, we are grateful to have become published authors confident in our research and in our abilities as scholars, scientists, and critical thinkers (*n* = 1999).

## Author contributions

Both authors worked collaboratively on this paper. SM and MR brainstormed together, and each author wrote sections of the paper. SM did the majority of editing, and both SM and MR proofread and made final edits to the manuscript.

### Conflict of interest statement

SM and MR worked in a research lab of one of the guest editors, Dr. Traci Giuliano.

## Appendix

Conference Presentations and Publications Resulting from Our 1-Year Lab Experience

**Undergraduate authors*
*Matthews, S. J., Giuliano, T. A., *Rosa, M. N., *Thomas, K. H., *Swift, B. A., *Ahearn, N. D., *Garcia, A. G., *Smith, S. R., *Niblett, C. M., & *Mills, M. M. (2018). The battle against bedroom boredom: Development and validation of a brief measure of sexual novelty in relationships. *Canadian Journal of Human Sexuality*. Advance online publication. doi: 10.3138/cjhs.2017-0041
*Matthews, S. J., Giuliano, T. A., *Rosa, M. N., *Thomas, K. H., & *Swift, B. A. (2018). Sexual Novelty Scale. Handbook of Sexuality-Related Measures. Thousand Oaks, CA: Sage Publications.
*Rosa, M. N., *Matthews, S. J., Giuliano, T. A., *Thomas, K. H., *Swift, B. A., & *Mills, M. M. (2018). Encouraging erotic variety: Identifying correlates of, and strategies for promoting, sexual novelty in romantic relationships. Manuscript submitted for publication.
*Thomas, K. H., *Rosa, M. N., *Swift, B. A., *Mills, M. M., *Matthews, S. J., & Giuliano, T. A. (2017). More than missionary: Predictors and correlates of sexual novelty in committed relationships. Poster presented at the annual meeting of the Society for the Scientific Study of Sexuality, Atlanta, November.
*Rosa, M. N., *Matthews, S. J., *Thomas, K. H., *Swift, B. A., *Mills, M. M., & Giuliano, T. A. (2017). Encouraging erotic variety: The effects of persuasion on attitudes toward sexual novelty. Poster presented at the annual meeting of the Society for the Scientific Study of Sexuality, Atlanta, November.
*Matthews, S. J., *Thomas, K. H., *Rosa, M. N., *Swift, B. A., *Mills, M. M., *Smith, S., *Niblett, C. M., *Ahearn, N. D., *Garcia, A. G., & Giuliano, T. A. (2017). Development and validation of a brief measure of sexual novelty in relationships. Poster presented at the annual meeting of the Association for Psychological Science, Boston, May.
